# Fabrication and Characterization of Micro-Nano Electrodes for Implantable BCI

**DOI:** 10.3390/mi10040242

**Published:** 2019-04-11

**Authors:** Ye Xi, Bowen Ji, Zhejun Guo, Wen Li, Jingquan Liu

**Affiliations:** 1 National Key Laboratory of Science and Technology on Micro/Nano Fabrication Laboratory, Department of Micro/Nano-electronics, Shanghai Jiao Tong University, Shanghai 200240, China; yesiki@sjtu.edu.cn (Y.X.); jibowen2015@sjtu.edu.cn (B.J.); gzj98762@sjtu.edu.cn (Z.G.); 2 Department of Electrical and Computer Engineering, College of Engineering, Michigan State University, East Lansing, MI 48823, USA

**Keywords:** micro/nano electrode, electrochemistry, modified electrode, implantable BCI

## Abstract

Signal recording and stimulation with high spatial and temporal resolution are of increasing interest with the development of implantable brain-computer interfaces (BCIs). However, implantable BCI technology still faces challenges in the biocompatibility and long-term stability of devices after implantation. Due to the cone structure, needle electrodes have advantages in the biocompatibility and stability as nerve recording electrodes. This paper develops the fabrication of Ag needle micro/nano electrodes with a laser-assisted pulling method and modifies the electrode surface by electrochemical oxidation. A significant impedance reduction of the modified Ag/AgCl electrodes compared to the Ag electrodes is demonstrated by the electrochemical impedance spectrum (EIS). Furthermore, the stability of modified Ag/AgCl electrodes is confirmed by cyclic voltammogram (CV) scanning. These findings suggest that these micro/nano electrodes have a great application prospect in neural interfaces.

## 1. Introduction

The multidisciplinary and multilevel comprehensive research on the higher cognitive function of the human brain has become one of the mainstream directions in contemporary scientific development [1,2,3,4,5,6,7,8,9]. Brain-computer interface (BCI) technology emerges in this condition which establishes a direct communication and control channel between the human brain and computers, or other electronic devices. Neuroscience studies show that electrical activity of the nervous system will have a corresponding change after the brain produces a motion of consciousness or receives certain outside stimulation. Through BCI technology, these neural signals can be recorded and converted into relevant command signals to drive external devices. One of the key aspects of BCI technology is the effective acquisition of physiological signals in neural activity through implantable electrodes [4]. Analyzing the bioelectrical signals collected by the electrodes will help us to comprehend brain mechanisms more clearly. Therefore, it is of great significance to develop implantable electrodes with novel structure and materials for BCI technology. For instance, Wang M. et al. successfully fabricated the microelectrodes modified with direct electrodeposition of composites for application in neural interfaces [8]. Wu, F. et al. prepared integrated µLEDs on silicon neural probes for a high-resolution optogenetic study [9]. However, the BCI technology still faces challenges in the biocompatibility and long-term implantation of devices after implantation [10,11,12,13,14]. Because of the mechanical mismatch between the material and the nerve tissue, the electrodes will be encapsulated by glial cells due to the chronic foreign body response after long-term implantation. Therefore, the electrode recording points will lose the function of accurately recording electrical signals. In addition, the device implantation can cause damage to the neurons which are in the same plane with, or near the recording points of the electrode, and the reliability of the measured bioelectrical signals will be also affected. Compared to electrodes with a structure similar to a flat plate, the damage to peripheral neurons caused by needle electrode implantation mainly occurs on the cone surface, which is not in the same plane with the electrode recording points. At the same time, the conical structure of needle electrodes can reduce damage to human tissue during the implantation [15,16]. 

A widespread method to fabricate needle micro/nano electrodes is the laser-assisted pulling method because of the excellent control of the electrode geometry and its high repeatability [17,18,19,20,21,22,23,24]. In this method, a tension is applied to both ends of the sample which consists of a glass tube and a metal wire inside it during the laser heating. During the process of laser heating and pulling, the plastic sample can be pulled down to a pair of independent metal wire tips trapped in glass with a smaller diameter. Then the tip is polished to expose the metal core. Except for the area of the electrode at the tip, the entire electrode is encased in a thin layer of glass. Multiple electrode recording points can be realized by pulling multi-barrel glass tubes, and the electrode array can also be realized by pulling multiple glass tubes together [23,24]. It can realize a multifunctional integration of electrodes through carbon pyrolysis, electroplating and modification of functional groups.

At present, common metal materials used in the laser-assisted pulling method mainly include Au and Pt. Due to the mismatch in physical properties, it is still difficult to prepare needle electrodes with metals other than Au and Pt [20]. Therefore, it is of great significance to develop a laser-assisted pulling method with other materials. In addition to excellent ductility and the highest electrical and thermal conductivity of all metals, silver also has good prospects in the medical field. Silver is widely used in biomedical hydrogels, bandages and long-term implanted devices because of its antibacterial properties and biocompatibility [25,26]. In this paper, the Ag needle micro/nano electrodes were fabricated. By optimizing preparation process and parameters, the mismatch of melting point and other physical properties between quartz and silver were successfully resolved, and the Ag/AgCl needle microelectrodes were fabricated by electrolytic chlorination. The Ag/AgCl electrode is close to the non-polarized electrode at low current and the current flowing, through the electrode during bioelectrical signal measurement is weak. Therefore, Ag/AgCl electrodes are suitable for the determination of bioelectrical signals as a detection electrode [27,28]. The electrochemical impedance spectrum (EIS) method and stability test were carried out to characterize the Ag/AgCl electrodes after modification. Finally, the preparation of a needle nano electrode is discussed, on the basis of the preparation of needle micro electrodes. The SEM images confirmed the successful preparation of the Ag needle nanoelectrode.

## 2. Materials and Methods 

### 2.1. Materials

Polyethylene glycol (PEG, 2000), potassium chloride (KCl), hydrochloric acid (HCl), acetone (CH_3_COCH_3_) and phosphate buffered saline (PBS, pH 7.4) were purchased from Sinopharm Chemical Reagent Company, Ltd. (Shanghai, China). All chemicals used in the experiments were of reagent grade quality or better and were used without further purification. Aqueous solutions were prepared from de-ionized water. Quartz capillaries (o.d. = 1.0 mm, i.d. = 0.3mm) were purchased from Sutter Instrument Company (Novato, CA, USA). 

### 2.2. Instruments

The laser-based micropipette puller system (P-2000, Sutter Instrument Company) was used in the fabrication of the Ag electrodes. High vacuum scanning electron microscopy (ULTRA55, Zeiss, Jena, Gemany) was used for SEM observation. The anodic oxidation process of Ag electrodes was carried out on a CHI 660c electrochemical workstation (CH Instruments, Austin, TX, USA). Other electrochemical measurements such as cyclic voltammogram (CV) and electrochemical impedance spectrum (EIS) were conducted by Autolab (PGSTAT204, Metrohm, Herisau, Switzerland). An electrode beveler (BV-10, Sutter Instrument Company) was used in the polishing process.

### 2.3. Preparation of the Ag Needle Electrode

**Figure 1:**
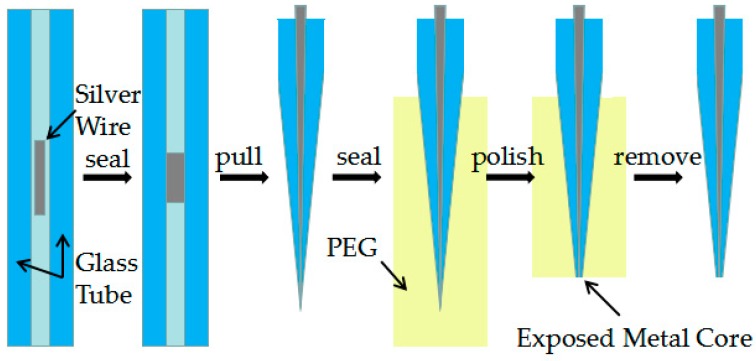
Process diagram showing the preparation of needle electrodes with the laser-assisted pulling method.

Figure 1 shows a five-step fabrication process of Ag needle electrodes. At first, silver wires were pretreated with fine grit sandpapers with different grits to remove oxides from the surface. Afterward they were treated with ultrasonic cleaning in acetone, ethanol and de-ionized water and dried in a drying oven at 200° Celsius, a ~2 mm long piece of silver wire was inserted into a 75 mm long piece of quartz capillary (o.d. = 1.0 mm, i.d. = 0.3mm). It is worth noting that the length of silver wire will directly influence the morphology of the needle electrode obtained by the laser-assisted pulling method under certain optimization parameters. 

**Figure 2:**
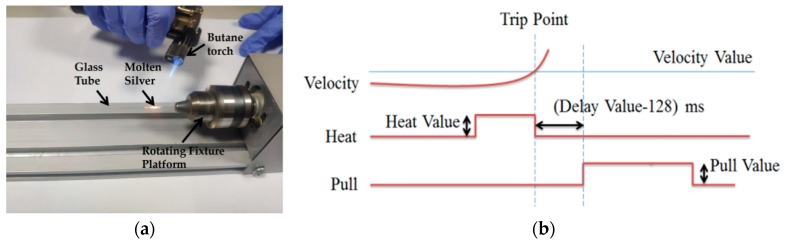
(**a**) Picture of the rotating fixture platform adopted in the melting process. (**b**) A typical pull cycle based on the P-2000 laser-based micropipette puller system.

In the second step, the silver wire was sealed in quartz glass by a butane torch. The temperature of the butane torch flame was about 1300° Celsius, which is between the melting temperature of the silver wire and the softening temperature of quartz glass. Heated by the butane torch, the silver melted and then adhered to the inner wall of the glass tube. As shown in Figure 2a, a rotating fixture platform was adopted in the melting process in order to obtain a better adhesion between the silver wire and quartz glass inner wall. After the melting process, the sealing was checked under an optical microscope to ensure that silver wire adhered to the inner wall well. 

In the third step, a laser-assisted pulling method was adopted to prepare the Ag needle electrodes. The P-2000 laser-based micropipette puller system was used in the pulling process. Figure 2b shows a typical pull cycle based on the P-2000 laser-based puller system. The adjustment parameters of the laser needle puller system were as follows: Heat, Velocity, Delay, Pull and Filament. The parameter Heat (range from 0 to 999) controls the power of the laser applied on the capillary glass tube. The typical range of Heat setting is around 700 to 999 for quartz. The parameter Velocity (range from 0 to 255) is a temperature-dependent parameter that determines the trip point at which the equipment stops laser heating. The sample will have a higher softening degree before hard pulling with an increase of the parameter Velocity. The parameter Delay (range from 0 to 255) determines the time interval between stopping laser heating and applying a pull. A change of one unit represents a change of 1 ms to the delay time. When the value of parameter Delay is 128, the equipment will stop heating and apply tension at the same time. If the parameter Delay is larger than 128, the hard pull will begin after the end of a laser heating. If the parameter Delay is smaller than 128, the hard pull will begin before the end of a laser heating. The parameter Pull (range from 0 to 255) determines the magnitude of tension applied at both ends of the glass tube. In general, the higher the parameter Pull, the smaller the tip of electrode will be prepared. The parameter Filament (range from 0 to 5) specifies the scan length of the laser beam which is used to heat the designated area of the glass tube. When the parameter Filament is set from 0 to 5, the corresponding scanning lengths are 1 mm, 1.5 mm, 1.9 mm, 4.5 mm, 6.5 mm and 8 mm, respectively. These five parameters influence and restrict each other. The sealing, containing quartz glass and silver wire, was pulled into two independent silver tips trapped in quartz glass under certain parameters. In this step, the morphology of the prepared needle electrode is significantly affected by different parameters.

In the fourth step, the tip portion was covered in PEG which is water-soluble. This operation was used to increase the rigidity of the ultrafine tip for subsequent polishing. The sample was roughly polished with sandpapers of different grits. By using a KCl solution containing alumina suspension on a polishing cloth, the silver tip was further exposed and sanded with the aid of a Sutter BV-10 electrode beveler which was situated just below a vertical micro-mobile platform. During the further polishing process, the sample was fixed on a micro-mobile platform with a displacement accuracy of 1 μm and the sample was polished with a decrease in the vertical direction. To control the exposure of the tip, the electrical resistance between the silver wire embedded in the composite material and the polishing cloth was tested after every certain depth of polishing through a multimeter [22]. Finally, the PEG wrapped around the outside of the entire tip was removed in hot liquids and a Ag electrode with a smooth surface was obtained.

### 2.4. Modification of the Ag Needle Electrode

The Ag electrode was cleaned with acetone and de-ionized water to remove surface contaminants. Then the electrode was anodized in 0.1 M HCl aqueous solution on a CHI 660c electrochemical workstation. The platinum electrode was used as a cathode and the Ag electrode was connected to the anode. The whole anodic oxidation process was under a constant voltage of 1 V. 

## 3. Results and Discussion

### 3.1. Fabrication of the Ag Needle Microelectrode

**Figure 3:**
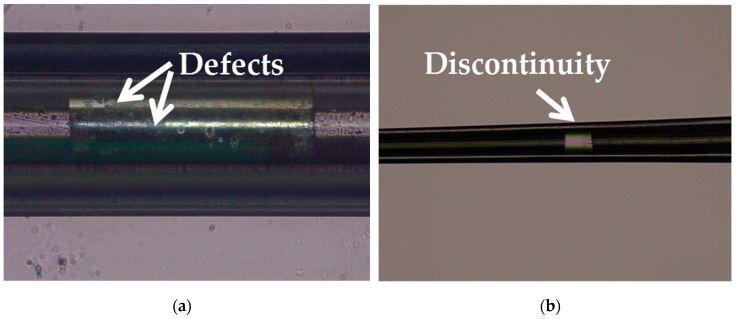
(**a**) Micrograph of the adhesion surface between the silver and the inner wall of the glass tube. (**b**) Micrograph of the discontinuity occurred in the internal silver wire.

**Figure 4:**
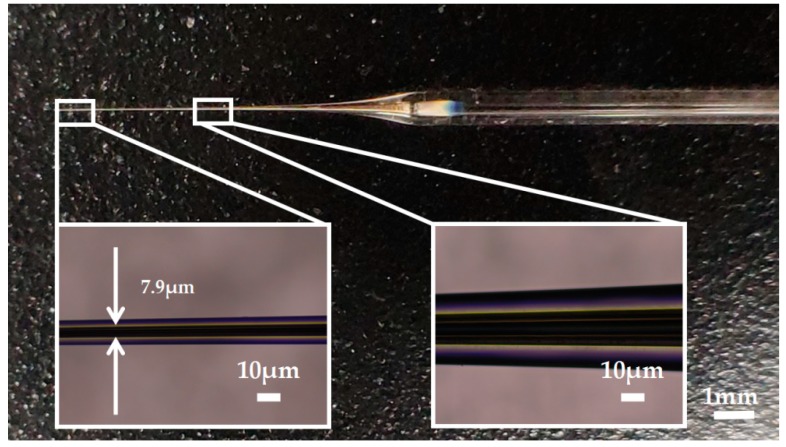
The picture of the Ag needle electrode.

Silver has a melting point of 961.78° Celsius which is far below the softening temperature of quartz glass. In the process of sealing the silver into the quartz glass tube with a butane torch, the silver melted first, while the quartz glass tube remained solid. The melted silver solidified and adhered to the inner wall of the quartz glass tube as the temperature decreased. The attached quality between silver and the inner wall of the glass tube would affect the morphology of prepared electrode. Figure 3a shows the adhesion surface between the silver and the inner wall of the glass tube. The adhesion surface was dotted with some defects, which may lead to the discontinuity of the internal silver wire as shown in Figure 3b. In order to improve the adhesion quality, a rotating fixture platform was adopted in the heating melting process. In the high-speed rotation, the silver was tightly attached to the inner wall of the glass tube. Through analysis and optimization of various parameters in laser-based puller system, the Ag needle electrode was successfully fabricated under the following parameters: Heat = 850, Filament = 4, Velocity = 30, Delay = 150 and Pull = 170. As shown in Figure 4, the results confirmed that the mismatch between quartz and silver was successfully resolved by optimizing preparation process and parameters. The effective recording point of the electrode tip was ~7.9 μm in diameter and the variable-diameter part of the needle electrode was ~9.2 mm in length. Micrographs showed that the needle electrode presented a good internal continuity and there was no cracks in the surrounding glass.

### 3.2. Electrochemical Characterization of the Needle Microelectrodes

By an anodic oxidation process, silver atoms were oxidized to silver ions at the electrode-electrolyte interface and then deposited on the electrode surface after combining with chloride ions. Equation (1) and (2) express the specific reaction process occurring in the anodic oxidation process [28]:

(1) $$\mathrm{Ag}↔{\mathrm{Ag}}^{+}+e$$

(2) $${\mathrm{Ag}}^{+}+Cl^{-}↔AgCl↓$$

**Figure 5:**
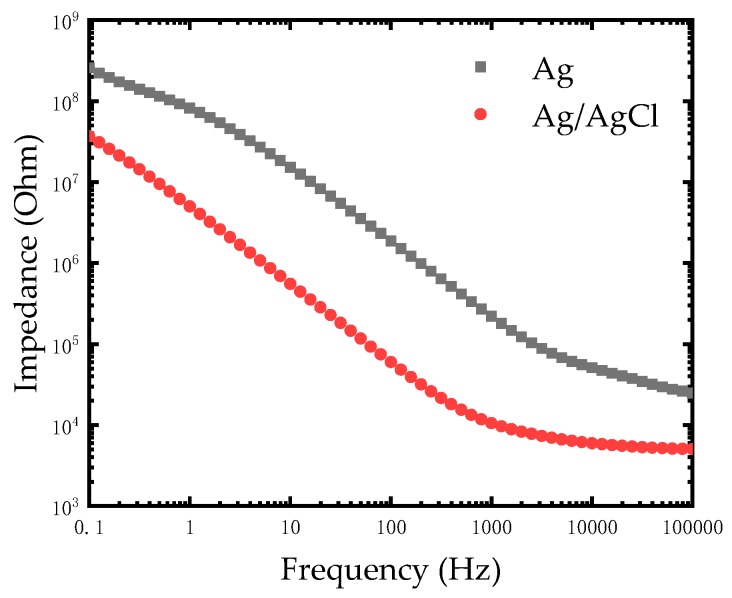
Impedance comparison diagram of the Ag electrode and modified Ag/AgCl electrode in phosphate buffered saline (PBS) solution.

As seen in Figure 5, the impedance of the modified Ag/AgCl electrode decreased compared to the Ag electrode at a low frequency from 0.1 Hz to 1000 Hz. The impedances of electrodes at low frequency are compared in Table 1. This experiments result was in agreement with the theory that silver chloride is considered as a type of non-polarizable material while silver is a polarizable material. Based on the excellent electrical conductivity of silver, the impedance of the modified Ag/AgCl electrode further reduced at low frequency and it was advantageous for measuring and recording bio-electricity signals. 

### 3.3. Stability Tests

**Figure 6:**
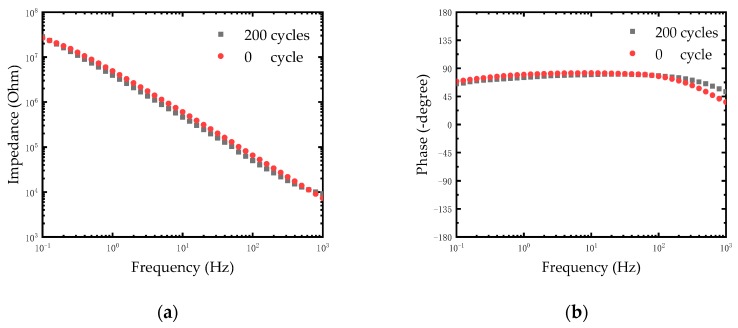
(**a**) Impedance plot of modified Ag/AgCl electrode treated with different cycles of CV scanning. (**b**) Phase spectra of modified Ag/AgCl electrode treated with different cycles of CV scanning.

After chlorination of Ag electrodes, a thin film of silver chloride was formed on the surface of the electrodes. The quality of the film would directly affect the stability of electrodes. The stability of the modified electrodes was evaluated by repetitive CV scanning. The CV scanning process was operated in PBS solution at room temperature. The Ag/AgCl electrode and the Pt electrode were used as anodes and cathodes respectively. We carried out 200 cycles of CV scanning with the scan rate of 1 V/s between -0.6 V and 0.8 V and the EIS of electrodes was measured. As shown in Figure 6a, the impedance of electrodes remained relatively stable after 200 cycles. The impedance of the electrode at 1 KHz was reduced by one fifth (from 9140.11 Ω to 7312.46 Ω). The reduction of impedance may be caused by further chlorination of silver on the electrode surface after rapid CV scanning. This is because the reaction of the Ag/AgCl electrode is an electrolytic reaction rather than a polarization of the metal. The following equilibrium reaction occurs on the electrode surface:

(3) $$\mathrm{AgCl}+e↔\mathrm{Ag}+{\mathrm{Cl}}^{-}$$

Therefore, the thickness of the AgCl layer changes periodically during the rapid CV scanning. The results in Figure 6b shows the phase of electrodes after 200 cycles. Considering that the typical electroencephalography (EEG) signal amplitude was less than 1 mV, the rapid CV scanning test equaled a destructive test to detect the stability of the Ag/AgCl electrode. The experimental results demonstrated that the modified Ag/AgCl electrodes performed with good stability, which could meet the neural signal measurement in a complex environment in vivo.

### 3.4. SEM Images Characterization of the Ag Needle Nanoelectrodes

**Figure 7:**
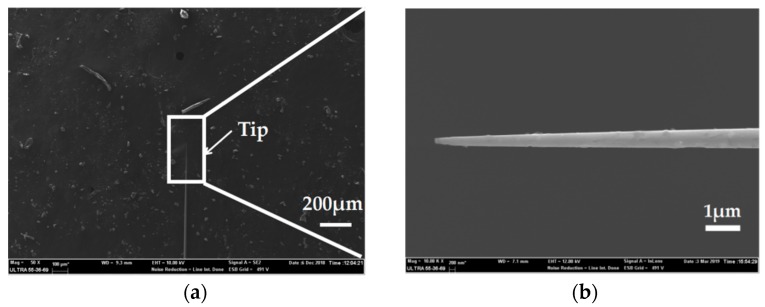
(**a**) SEM image of the entire silver/glass tip of the Ag nanoelectrode. (**b**) SEM image with an enlarged scale of the silver/glass tip of the Ag nanoelectrode.

For microelectrode preparation, we fabricated the needle electrodes at nano-scale which had the ability to record signals in a single neuron cell due to its great spatial resolution. SEM characterization was carried out to observe the morphology of the Ag needle submicron electrodes. Figure 7a shows a SEM image of an entire silver/glass tip after the pulling process under the following parameters: Heat = 910, Filament = 2, Velocity = 30, Delay = 128 and Pull = 250. Compared to the parameters in the preparation of the microelectrodes, the increase of laser power and the decrease of scan length play significant roles in the reduction of electrode size. A SEM image with an enlarged scale of a silver/glass tip is shown in Figure 7b. Because the diameter ratio between the quartz and the silver wire was consistent with the initial ratio between the quartz tube and the silver wire [21], the diameter of the Ag electrode was less than 100 nm. This result confirmed a successful preparation of Ag needle electrodes at the nano-scale.

## 4. Conclusions

In conclusion, we developed the fabrication of Ag needle micro/nano electrodes with a laser-assisted pulling method. Through analyzing and optimizing the parameters in laser-based puller system, the Ag needle microelectrodes with a diameter of 7.9 μm were successfully fabricated. An anodic oxidation process which converted silver to silver chloride was operated to modify the electrode surface. The electrochemistry performance of the modified Ag/AgCl electrode was researched by studying the EIS. After modification, the impedance of electrode at 1 KHz changed from 2.2 × 10^5^ Ω to 1.05 × 10^4^ Ω. The results showed that the impedance of the modified Ag/AgCl electrodes presented a significant reduction after surface modification. The stability of modified Ag/AgCl electrodes was also verified by CV scanning. On the basis of the microelectrode preparation, we prepared the needle electrodes at the nano-scale which can further improve the spatial resolution of the device. These findings suggest that these micro/nano electrodes have great application prospects in neural interfaces. In future work, we will continue to focus on electrode scaling reduction and single cell signal acquisition. 

## Figures and Tables

**Table 1:** The impedances of the Ag electrode and modified Ag/AgCl electrode at different frequencies.

Frequency (Hz)	The Ag Electrode (Ω)	The Ag/AgCl Electrode (Ω)
1000	2.2 × 10^5^	1.05 × 10^4^
100	1.87 × 10^6^	6.01 × 10^4^
10	1.52 × 10^7^	5.56 × 10^5^
1	8.19 × 10^7^	5.01 × 10^6^
0.1	2.56 × 10^8^	3.69 × 10^7^
